# The Effect of Repair Materials and Surface Treatments on the Shear Bond Strength of 3D-Printed Provisional Restoration

**DOI:** 10.1055/s-0044-1791979

**Published:** 2025-03-12

**Authors:** Pattaramon Hanpongkittikun, Wissanee Jia-mahasap, Boonchai Chaoklaiwong

**Affiliations:** 1 Department of Prosthodontics, Faculty of Dentistry, Chiang Mai University, Chiang Mai, Thailand.

**Keywords:** shear bond strength, 3D printing, interim dental prosthesis, CAD-CAM

## Abstract

**Objective** Despite the emergence of numerous three-dimensional (3D) printed provisional resin, there are no conclusive guidelines for repairing them. This study aims to investigate the effects of different repair materials and surface treatments on the shear bond strength of 3D-printed provisional resin.

**Materials and Methods** A total of 180 3D-printed resin specimens underwent six surface treatments: no surface treatment (control), silicon carbide paper (SP), sandblasting with aluminum oxide (SB), SP followed by SB (SP + SB), SP with bonding agent (SP + BD), and SB with bonding agent (SB + BD). Each group was repaired with polymethyl methacrylate (PMMA), Bis-acryl, and flowable composite resin (FCR). The shear bond strength of the bonded specimens was tested using a universal testing machine and the mode of failure was examined with stereomicroscope.

**Statistical Analysis** The Shapiro–Wilk test was used to assess normal distribution, and two-way analysis of variance (ANOVA; *α* = 0.05) was used to find the effect of independent variables on the shear bond strength. The post hoc test was achieved using the Tukey honest significant difference (HSD) test.

**Results** Two-way ANOVA indicated a statistically significant interaction between repair materials and surface treatments in relation to the shear bond strength of 3D-printed resin ( *p* < 0.001). The three highest shear bond strengths overall were SB repaired with Bis-acryl (17.30 ± 0.77 MPa), SB + BD repaired with FCR (17.20 ± 0.29 MPa), and SB + BD repaired with PMMA (16.60 ± 0.71 MPa), which were significantly higher than their control group ( *p* < 0.001). However, there were no significant differences between these groups. Notably the lowest shear bond strength in PMMA occurred in the control group (8.49 ± 0.42 MPa), while the lowest shear bond strength in Bis-acryl and FCR was observed in the SP group (7.28 ± 0.71 and 8.84 ± 1.15 MPa, respectively).

**Conclusion** Repair materials and surface treatments play an important role in repairing 3D-printed resin. PMMA and composite resin–based repair materials require both sandblast and a chemical bonding agent, while Bis-acryl-based repair materials only need sandblasting to improve the shear bond strength.

## Introduction

Digital prosthetic dentistry has incorporated the use of computer-aided design and computer-aided manufacturing (CAD/CAM) technology to help with the fabrication of dental prostheses. Additive manufacturing, which is also known as three-dimensional (3D) printing, has been developed to enable the formation of more complex structures and simultaneous production of numerous subjects. In addition, 3D printing is considered more cost-effective than subtractive manufacturing. 1 There are many additive manufacturing processes; however, only certain methods are widely used in dentistry, including powder bed fusion (PBF), binder jetting (BJT), material jetting (MJT), material extrusion (MEX), and vat photopolymerization (VPP), while others are more prevalent in different industries. PBF is also known as selective laser sintering and laser melting, which are used for producing metal such as Co-Cr/titanium framework and metal crown. 1 2 3 BJT, MJT, and MEX are frequently used for model and surgical guide. 1 2 3 One of the most commonly used 3D printing technologies in dentistry is VPP, which includes stereolithography (SLA) and digital light processing (DLP). Both SLA and DLP employ photopolymerization techniques that produce smooth surfaces and fine details, making them ideal for creating models, castables, surgical guides, splints, trays, gingival masks, dentures, and provisional restorations. 1 2 3 4 Provisional material selection should be based on mechanical, physical, and handling properties. The material should be biocompatible; nonirritating to oral tissue; exhibit good marginal adaptation, high tensile strength, and dimensional stability; and aesthetically acceptable and reparable. 5 For intraoral use, 3D-printed provisional restorative materials offer adequate mechanical properties, 4 with some studies indicating superior mechanical properties compared to CAD/CAM milled and other conventionally fabricated options. 6 7 As a result, they can be used as an alternative to conventional and CAD/CAM milled long-term provisional materials. 7 However, 3D-printed provisional restorations could break and require repair when used for a long period. The choice of repair material and surface treatment significantly influences the bond strength of restorative works. 5 Materials often used for chairside repair provisional restoration are autopolymerized acrylic resin or polymethyl methacrylate (PMMA), Bis-acryl, and flowable composite resin (FCR) or bisphenol A-glycidyl methacrylate (Bis-GMA). 5 8 Autopolymerized acrylic resin gives quick and simple manipulation; however, it also presents with odor, shrinkage, and heat production; Bis-acryl composite resin provides low exothermic reaction with minimal shrinkage; and light curing FCR offers low polymerization shrinkage with minimal odor. 5 For 3D-printed provisional resin, various research studies have addressed this issue with diverse approaches and outcomes. Lim and Shin 9 suggested using Bis-acryl composite without additional surface treatment. Jeong and Kim 10 found that Bis-acrylic material yielded optimal results when coupled with surface treatment involving silicon carbide paper and sandblasting. However, Albahri et al 11 observed no statistically significant differences in shear bond strength among PMMA, Bis-acryl, and composite resin. Nevertheless, they emphasized the predictability of outcomes when using composite resin due to fractography analysis. Palavicini et al 12 demonstrated that relining 3D-printed provisional resin with conventional PMMA resulted in shear bond strength comparable to that achieved by relining PMMA base with conventional PMMA. From these studies, few compared three different repair materials, specifically PMMA, Bis-acrylic, and composite resin. Furthermore, there is no consensus of recommendation for the best material suitable for repairing 3D-printed provisional restoration and the optimal protocol for surface treatment of 3D-printed provisional restoration prior to repair. The purpose of the present study is to investigate the effects of different repair materials and surface treatments on the shear bond strength of 3D-printed provisional resin. The null hypothesis was there would be no significant difference in shear bond strength among different repair materials and different surface treatments of 3D-printed resin.

## Material and Methods

Sample size calculation was performed using the G*Power software (version 3.1.9.7; University of Heinrich-Heine, Düsseldorf, Germany) based on our pilot study of the effect size (1.725), alpha of 0.05, and 80% power of the test. This calculation yielded a sample size of at least two samples per group.

Cylindrical shape 3D-printed resin samples ( *n* = 180) with a diameter of 8 mm and a height of 12 mm were made from provisional resin (Temporary CB; Formlabs Inc., Somerville, MA, United States) using a 3D printing machine (Formlabs SLA 3B; Formlabs Inc.). They were printed at a 0-degree angle with a layer thickness of 50 µm. After printing, they were washed in isopropyl alcohol (Form Wash; Formlabs Inc.) at a concentration of ≥99% for 3 minutes, followed by postcuring (Form Cure; Formlabs Inc.). During postcuring, the resin samples were placed with the raft side down and the support intact at 60°C for 20 minutes. After removing the support, they were postcured at 60°C with the other side up for an additional 20 minutes. The 3D-printed resin samples were categorized into six groups for different surface treatments ( *n* = 30).

- Group 1 (C): Control with no surface treatment.
- Group 2 (SP): Treated with 220-grit silicon carbide paper using a grinding machine (LaboPol-20; Struers, Ballerup, Denmark) then ultrasonic cleaning (Easyclean; Renfert, Hilzingen, Germany) in distilled water for 5 minutes.
- Group 3 (SB): Sandblasted with 50-µm aluminum oxide (Basic eco; Renfert, Hilzingen, Germany) 10 mm, 0.2 MPa, 10 seconds, then ultrasonic cleaning in distilled water for 5 minutes.
- Group 4 (SP + SB): Treated with 220-grit silicon carbide paper and sandblasted with 50-µm aluminum oxide, 10 mm, 0.2 MPa, 10 seconds, then ultrasonic cleaning in distilled water for 5 minutes.
- Group 5 (SP + BD): Treated with 220-grit silicon carbide paper and then ultrasonic cleaning in distilled water for 5 minutes. After that, apply bonding (single bond universal adhesive; 3M ESPE, St. Paul, MN, United States), scrub for 20 seconds, air dry for 5 seconds, and light cure for 10 seconds.
- Group 6 (SB + BD): Sandblasted with 50-µm aluminum oxide, 10 mm, 0.2 MPa, 10 seconds, then ultrasonic cleaning in distilled water for 5 minutes. After that, apply bonding, scrub for 20 seconds, air dry for 5 seconds, and light cure for 10 seconds.

The surface texture of 3D-printed resin was examined with a scanning electron microscope (SEM; JSM IT800, Joel Ltd., Akishima, Tokyo) under ×1,000 magnification.

**Fig. 1:**
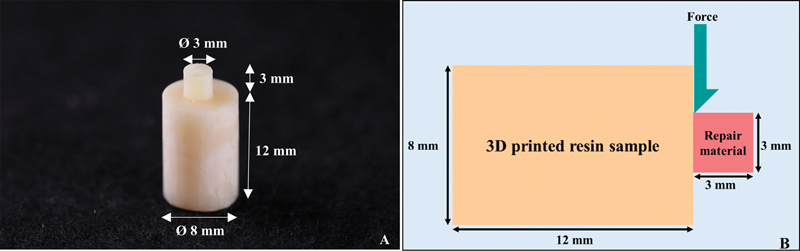
( **A** ) Dimension of the specimen, bonded specimen. ( **B** ) Schematic diagram of the shear bond strength test conducted on the bonded 3D-printed resin sample with repair material. 3D, three-dimensional.

Each surface treatment group was repaired with PMMA (UNIFAST Trad; GC America), Bis-acryl (Protemp 4; 3M ESPE) and FCR (Filtek Supreme Flowable Restorative; 3M ESPE) by using a metal mold with a diameter of 3 mm and height of 3 mm ( *n* = 10). PMMA was mixed according to the manufacturer's instructions and injected into the mold with a plastic syringe. Bis-acryl was mixed using an auto-mixing gun and injected into the mold. Glass slap was placed on top to remove the excess material. The repair materials were allowed to set at room temperature for 60 minutes. FCR was injected into the mold and a glass slap was placed on top to remove the excess material, followed by light curing with light-emitting diode (LED) light with a minimum intensity of 550 mW/cm ^2^ in the 400- to 500-nm range for 40 seconds. The bonded specimen ( Fig. 1A ) was stored in distilled water at 37°C for 24 hours. The shear bond strength was tested using a universal testing machine (Instron 5566; Instron, Norwood, MA, United States) with a crosshead speed of 0.5 mm/min until the repair material dislodged from 3D-printed resin ( Fig. 1B ). The shear bond strength (MPa) was determined by dividing the fracture load (N) by the bonded interface area (mm ^2^ ).

**Fig. 2:**
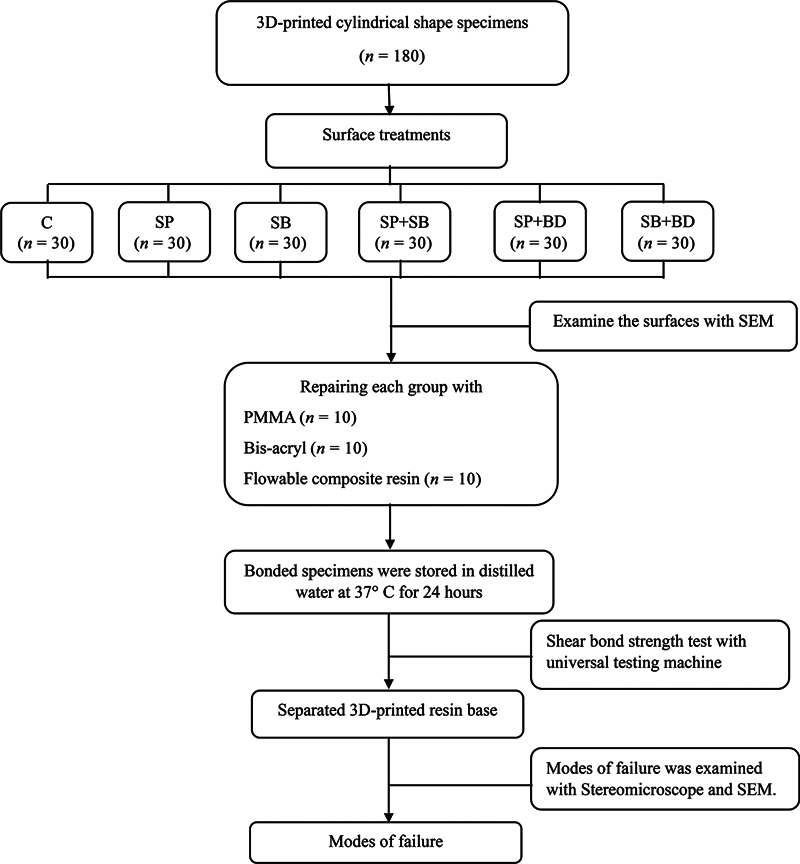
Flowchart showing the overall research methodology. 3D, three-dimensional; PMMA, polymethyl methacrylate; SEM, scanning electron microscope. C, control; BD, bonding; SB, sandblast; SP, silicon carbide paper.

The separated surface was examined with a stereomicroscope system and digital camera (SZX7 & SZ2-ILST led illuminator stand & E-330; Olympus, Tokyo, Japan) and SEM to assess the mode of failures under the following criteria: A provisional base crack exceeding 50% was considered a cohesive failure, while a repair material that remained less than 10% was considered an adhesive failure, and failure that was intermediate between the two failure modes was considered a mixed failure. 10 The flowchart of the overall methodology is shown in Fig. 2 .

Statistical analyses were performed by using a statistical software program (IBM SPSS statistics version 26.0; IBM Corp., Armonk, NY, United States). The Shapiro–Wilk test was used to assess normal distribution, and two-way analysis of variance (ANOVA; *α* = 0.05) was used to find the effect of independent variables on the shear bond strength. The post hoc test was achieved using the Tukey honest significant difference (HSD) test.

## Results

Two-way ANOVA indicated a statistically significant interaction between repair materials and surface treatments in relation to the shear bond strength of 3D-printed resin ( *p* < 0.001) as shown in Table 1 . The shear bond strength (Mpa) of each group with multiple comparison is shown in Table 2 . The three highest shear bond strengths overall were SB repaired with Bis-acryl (17.30 ± 0.77 MPa), SB + BD repaired with FCR (17.20 ± 0.29 MPa), and SB + BD repaired with PMMA (16.60 ± 0.71 MPa), which were significantly higher than their control group ( *p* < 0.001). However, there were no significant differences between these groups. Notably the lowest shear bond strength in PMMA occurred in the control group (8.49 ± 0.42 MPa), while the lowest shear bond strength in Bis-acryl and FCR was observed in the SP group (7.28 ± 0.71 and 8.84 ± 1.15 MPa, respectively).

**Table 1:** Two-way ANOVA showed influence of repair materials and surface treatments on the shear bond strength of 3D-printed provisional resin

Source of variation	df	*F*	*p*
Surface treatment	5	43.736	<0.001
Repair material	2	8.960	<0.001
Surface treatment × repair material	10	17.009	<0.001

Abbreviations: ANOVA, analysis of variance; 3D, three-dimensional.

**Table 2:** Mean shear bond strength (Mpa) with standard deviations of each group

Materials	C	SP	SB	SP + SB	SP + BD	SB + BD
PMMA	8.49 ± 0.42 ^ab^	12.28 ± 0.68 ^cde^	13.58 ± 0.51 ^cdef^	13.96 ± 0.78 ^defg^	14.80 ± 0.38 ^efg^	16.60 ± 0.71 ^fg^
Bis-acryl	8.62 ± 0.42 ^ab^	7.28 ± 0.71 ^a^	17.30 ± 0.77 ^g^	14.35 ± 0.82 ^efg^	8.36 ± 0.58 ^ab^	13.88 ± 0.88 ^defg^
FCR	10.44 ± 0.98 ^abc^	8.84 ± 1.15 ^ab^	10.75 ± 0.51 ^bcd^	10.24 ± 0.47 ^abc^	16.04 ± 0.60 ^fg^	17.20 ± 0.29 ^g^

Abbreviations: C, control; BD, bonding; FCR, flowable composite resin; PMMA, polymethyl methacrylate; SB, sandblast; SP, silicon carbide paper.

Note: Superscripts represent multiple comparisons, where the same letter indicates that there were no statistically significant differences in values ( *p* > 0.05).

**Fig. 3:**
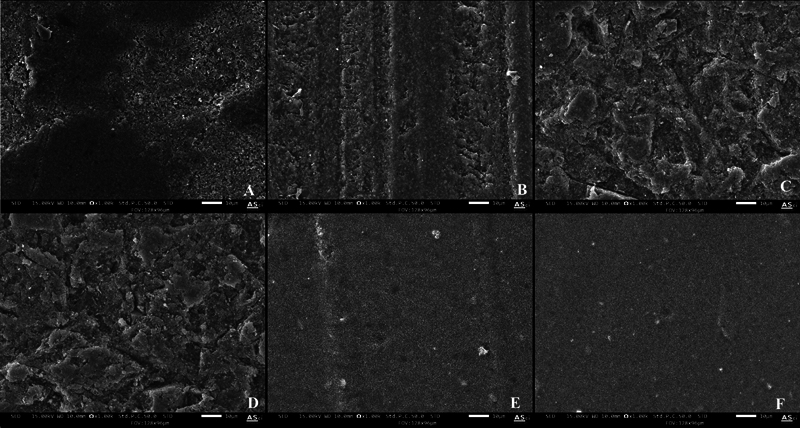
Scanning electron microscope images of 3D-printed resin after surface treatments using magnification ×1,000. ( **A** ) Control group. ( **B** ) SP group. ( **C** ) SB group. ( **D** ) SP + SB group. ( **E** ) SP + BD group. ( **F** ) SB + BD group. 3D, three-dimensional; BD, bonding; SB, sandblast; SP, silicon carbide paper.

SEM images ( Fig. 3 ) revealed distinct surface topographies of 3D-printed resin before repair. In the control group ( Fig. 3A ), an irregular surface was observed in the newly printed 3D resin. The SP group ( Fig. 3B ) displayed long grooves in a single direction, while the SB group ( Fig. 3C ) exhibited a rough surface with partial deep pits. The SP + SB group ( Fig. 3D ) demonstrated a surface topography similar to that of the SB group. The SP + BD group ( Fig. 3E ) displayed a smooth surface with bands, and the SB + BD group ( Fig. 3F ) consistently showed a smooth surface.

**Fig. 4:**
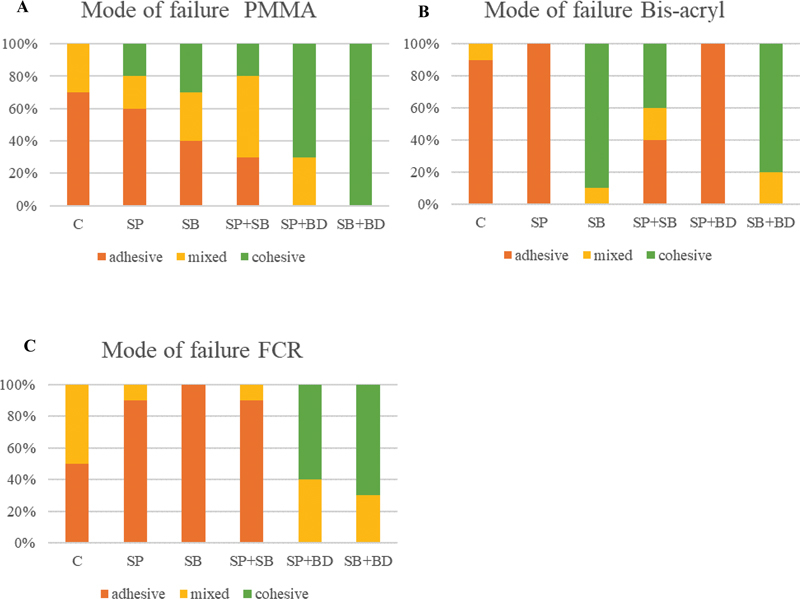
Mode of failure in percentage for each material. ( **A** ) PMMA. ( **B** ) Bis-acryl. ( **C** ) FCR. C, control; BD, bonding; FCR, flowable composite resin; PMMA, polymethyl methacrylate; SB, sandblast; SP, silicon carbide paper.

**Fig. 5:**
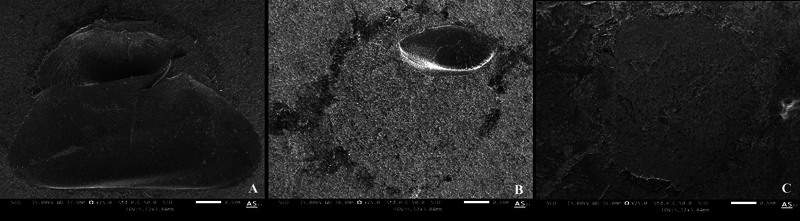
Scanning electron microscope images (×25) showing mode of failure for ( **A** ) cohesive failure, ( **B** ) mixed failure, and ( **C** ) adhesive failure.

The mode of failure within the PMMA repair material ( Fig. 4A ) showed mostly adhesive failure in the control surface treatment and presented with 70 to 100% cohesive failure in the SP + BD and SB + BD groups. Repairing with Bis-acryl ( Fig. 4B ) also predominated with adhesive failure in the control, SP, and SP + BD groups. Cohesive failure was observed when the surface was treated by the SB and SB + BD groups. With the FCR repair material ( Fig. 4C ), adhesive and mixed failures were distributed equally in the control surface treatment group, while the group that prepared the surface with SP + BD and SB + BD presented 60 to 70% cohesive failure. Fig. 5 showed SEM images of three different modes of failure.

## Discussion

In this study, the influence of three repair materials and six surface treatments on the shear bond strength of 3D-printed provisional resin was observed. According to the two-way ANOVA, a significant difference ( *p* < 0.001) has been found between the interaction of the two variables. Therefore, the null hypothesis was rejected.

Three chairside repair materials commonly utilized in dental clinics including autopolymerized acrylic resin, Bis-acryl composite, and flowable composite resin were selected. 5 UNIFAST Trad is mainly compose of PMMA, 13 while Protemp 4 is composed of functional methacrylate acid esters, 14 and Filtek Supreme Flowable Restorative is mainly composed of Procrylat, Bis-GMA, and TEGDMA. 15 The difference in chemical composition of the materials results in diverse adhesive properties and influences bonding to provisional material. 8

In the control surface treatment group, no statistically significant variation ( *p* > 0.05) was found in the shear bond strength of three different materials. However, the shear bond strength was significantly different upon mechanical and chemical surface treatments. Increasing surface roughness to promote mechanical retention and applying a bonding agent to encourage chemical bonding are two ways to strengthen the bond strength for repair materials. 5 9 10 16 17 Grinding with 220-grit silicon carbide paper and sandblasting with 50-µm aluminum oxide were the mechanical surface treatments used in this study. Grinding with silicon carbide paper represented using a carbide bur clinically, where it was simpler to manage the consistency and roughness. 10 As seen in Fig. 3B , the SP group showed long grooves on the surface of the 3D-printed surface, which produced the macroretentive effect. Sandblasting with 50-µm aluminum oxide roughened the surface and formed deep pits, as seen in Fig. 3C , which created a microretentive effect. 16 17 This study found that the shear bond strength was higher when preparing the surface with the SB group than with the SP group, indicating that the pattern of surface treatment could influence shear bond strength. As mentioned in other studies, micromechanical retention from sandblasting improved the capability of Bis-acryl resin to interlock mechanically to the old resin. 17 However, Lim and Shin 9 found that air abrasion alone was not enough to improve the bond strength. Another study found that roughening the PMMA surface solely through mechanical treatment did not provide an efficient bond between the repair surfaces and autopolymerized acrylic resin, indicating the need for additional chemical treatment. 5 18

In a previous study, repairing with Bis-acryl and surface treatment using silicon carbide paper conjugated with sandblasting gave the best result because of micromechanical retention and macromechanical retention. 10 This study found that the surface prepared with sandblasting demonstrated the best result for Bis-acryl but not significantly higher than the surface prepared with the SP + SB group. This might be because there was not enough roughness when preparing the first step with the 220-grit silicon carbide paper, causing the macroroughness to disappear after sandblasting. As seen in Fig. 3D , it showed a similar surface topography to the SB group.

For chemical surface treatment, a bonding agent was utilized in this study. It has been reported previously that the utilization of adhesive monomers significantly increases the repair bond strength in the filling composite resins. The bond strength between PMMA and FCR can be increased by first wetting the surface with a bonding agent to promote more efficient chemical bond between the surfaces. 16 Moreover, using a low-viscosity repair composite material with a bonding agent also enhances bond strength. 19 When the surface of 3D-printed resin in this study was mechanically treated with sandblasting and then wetting with a bonding agent (SB + BD), the shear bond strength of FCR was significantly increased than other mechanical surface treatment groups and cohesive failure occurred. Greater improvement of shear bond strength is expected from microretentive features and the infiltration of the resin into microscopic surfaces. 5 16 20 Revilla-León et al 21 also observed that 3D-printed dental provisional materials present similar composite bond strength to conventional materials, which provide favorable properties for composite repair and modifications. Albahri et al 11 also found that the group repaired with composite resin had more predictable outcome due to fractography analysis.

It was observed that the treatment of 3D-printed resin surface with bonding agent, followed by repairing with either PMMA or FCR, resulted in cohesive failures. This outcome implies that the application of bonding agent enhances the bond strength of these two materials. Conversely, in the case of Bis-acryl, it was ascertained that the utilization of bonding agent led to a decrease in bond strength when compared to the scenario where the surface was prepared solely with sandblasting. Jeong and Kim 10 found that the bond between Bis-acryl and 3D-printed resin did not occur when the bonding agent was applied, which could be attributed to a single bond universal having low pH. Furthermore, there was little inorganic filler content in 3D-printed resin to react with silane, and the presence of hydroxyethyl methacrylate (HEMA) may contribute to reduced bond strength due to its water sorption properties. 8

In this study, a bonding agent containing silane was used because silane contains an organofunctional group that reacts and copolymerizes with methacrylate groups. Its other end was a hydrolysable alkoxyl group that, once activated, could bond to an inorganic substrate. 22 In addition, Temporary CB, 3D-printed base material, contained silanized dental glass and inorganic filler, 23 which could create a stronger bond with silane. In contrast, Jeong and Kim 10 found that both general bonding and bonding containing silane did not affect the bond strength of the 3D-printed resin. In addition, it has been reported that the siloxane bond would be hydrolyzed by water at the interface over time and reduce the bond strength. 22 24

Multiple step reparation might be complicated to practice in clinical setting. Moreover, the clinical implications could depend on the resources available at each clinic. Repair materials are best used in controlled moisture environments, which implies that extraoral repair is preferable for controlling moisture. If intraoral repair is needed, sandblasting might require isolation with a rubber dam and high-volume evacuation devices for tissue protection, which lead to more complicated method. 25

The limitations of this study are that only one type of 3D-printed provisional material by SLA method was examined, which cannot be generalized to other materials, and it should be noted that the new 3D-printed resin materials are continuously being developed each year. Furthermore, it is crucial to acknowledge that oral environments may potentially yield different effects on the bond strength of 3D-printed resin overtime. Future studies should place greater emphasis on the multiple materials available in the market and compare their repair bond strength with other CAD/CAM methods. Moreover, these studies should focus on replicating the oral environment and assessing the long-term durability of the repair bond strength.

## Conclusion

The shear bond strength of 3D-printed provisional resin is influenced by repair materials and surface treatments. This *in vitro* study suggests that using a carbide bur alone might not be enough to strengthen the shear bond of 3D-printed provisional resin. Both sandblasting and chemical bonding agents are required to enhance the shear bond strength of PMMA and composite resin–based repair materials. Regarding Bis-acryl-based repair materials, sandblasting alone has been proven effective in improving shear bond strength.
